# Chronic fetal exposure to caffeine altered resistance vessel functions via RyRs-BK_Ca_ down-regulation in rat offspring

**DOI:** 10.1038/srep13225

**Published:** 2015-08-17

**Authors:** Na Li, Yongmei Li, Qinqin Gao, Dawei Li, Jiaqi Tang, Miao Sun, Pengjie Zhang, Bailin Liu, Caiping Mao, Zhice Xu

**Affiliations:** 1 Institute for Fetology, First Hospital of Soochow University, Suzhou, China; 2 Center for Perinatal Biology, Loma Linda University, California, USA

## Abstract

Caffeine modifies vascular/cardiac contractility. Embryonic exposure to caffeine altered cardiac functions in offspring. This study determined chronic influence of prenatal caffeine on vessel functions in offspring. Pregnant Sprague-Dawley rats (5-month-old) were exposed to high dose of caffeine, their offspring (5-month-old) were tested for vascular functions in mesenteric arteries (MA) and ion channel activities in smooth muscle cells. Prenatal exposure to caffeine increased pressor responses and vasoconstrictions to phenylephrine, accompanied by enhanced membrane depolarization. Large conductance Ca2^+^-activated K^+^ (BK_Ca_) channels in buffering phenylephrine-induced vasoconstrictions was decreased, whole cell BK_Ca_ currents and spontaneous transient outward currents (STOCs) were decreased. Single channel recordings revealed reduced voltage/Ca^2+^ sensitivity of BK_Ca_ channels. BK_Ca_ α-subunit expression was unchanged, BK_Ca_ β1-subunit and sensitivity of BK_Ca_ to tamoxifen were reduced in the caffeine offspring as altered biophysical properties of BK_Ca_ in the MA. Simultaneous [Ca^2+^]_i_ fluorescence and vasoconstriction testing showed reduced Ca^2+^, leading to diminished BK_Ca_ activation via ryanodine receptor Ca^2+^ release channels (RyRs), causing enhanced vascular tone. Reduced RyR1 was greater than that of RyR3. The results suggest that the altered STOCs activity in the caffeine offspring could attribute to down-regulation of RyRs-BK_Ca_, providing new information for further understanding increased risks of hypertension in developmental origins.

Since the concept of fetal origins of adult health/diseases was introduced in 1980 s^1^, increasing evidence have demonstrated that alteration of prenatal development increased risks of cardiovascular diseases in life later. Caffeine, a xanthine alkaloid, widely used in form of coffee or tea, is consumed by 68–74% of pregnant women at an average intake of 125–193 mg/day^2^. In pregnant women and rats, caffeine absorption was complete^3^, readily crossing placental barrier, and accumulated in fetal tissues^4^,^5^. Recent studies demonstrated that *in utero* exposure to maternal caffeine was associated with embryo toxicities^6^,^7^. Despite previous studies showed caffeine-affected cardiovascular disorders^8^, there has been very limited information on whether and how exposure to caffeine *in utero* may impact on pressor responses and development of hypertension.

A recent study demonstrated that malnutrition during pregnancy altered pressor responses and vascular activity associated with functional changes in Large conductance Ca^2+^-activated K^+^ (BK_Ca_) channels in the rat offspring^9^. Activation of vascular BK_Ca_ channels is an important buffering mechanism to counteract vessel depolarization and constriction. Activation of BK_Ca_ channel in the cell membrane allows K^+^ to flux out of the cell, causing hyper-polarization and consequent inhibition of voltage-dependent Ca^2+^ channels (VDCCs), resulting in vascular relaxation^10^. The coordination of BK_Ca_ and VDCCs plays an important role in membrane potential regulation and vasomotion.

It is rational that high concentrations of daily consumption of caffeine, instead of lower concentrations, are more likely to cause health concerns for under-matured fetuses. Thus, we designed a protocol of chronically using 20 mg/kg caffeine, twice a day, for a total caffeine of 40 mg/kg/day over almost whole pregnancy in rats. Based on the dose-conversion correlation between humans and rats (human: rats = 1:6.17), 40 mg/kg/day roughly equivalent to 4 cups of coffee (a cup of coffee contains nearly 150 mg of caffeine on average)^11^.

In hypothesizing that the prenatal insult may affect vascular functions related to ion channel mechanisms, the present study was designed to investigate following questions: Did chronic prenatal exposure to high concentrations of caffeine increase risks in development of vascular diseases in life later? Whether and how ion channels were involved in prenatal caffeine mediated-impact on vascular dysfunction?

## Results

### Body weight and phenylephrine-increased pressor responses

**Figure 1:**
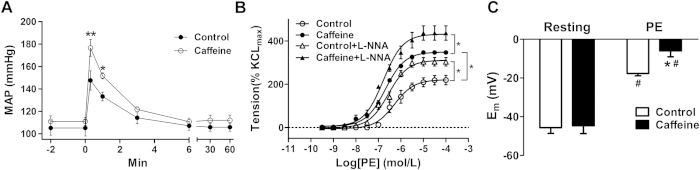
The effect of prenatal caffeine on phenylephrine (PE)-mediated pressor responses, vasoconstrictions, and membrane depolarization in offspring mesenteric arteries (MA). (**A**) Mean arterial pressure (MAP) in response to PE (n = 8 each group). 0 min: time for injection of PE. (**B**) Cumulative dose-response contractions in the MA induced by PE in absence or presence of L-NNA (10^−5^ mol/L) (n = 8 each group). (**C**) Depolarization of MA myocytes by 10^−5^ mol/L PE (n = 14 cells, 6 animals/each group). *P < 0.05, control vs. caffeine; ^#^p < 0.05, comparison for resting membrane potentials in the same group.

Prenatal caffeine (20 mg/kg, twice/day) significantly decreased fetal body weight to 86.73% compared to the control, with 25.1% of intrauterine growth restriction (IUGR) (Figure S1A). However, there were no significant differences in body weight in the adult offspring at 5-month-old (Figure S1B). As shown in Fig. 1A, phenylephrine-stimulated pressor responses was higher in the offspring exposed to prenatal caffeine than that of the control.

### Phenylephrine-increased vessel contractions and SMC depolarization in the offspring

In absence of the NO synthase inhibitor N^G^-nitro-L-arginine (L-NNA), the maximal response and pD_2_ values of PE-induced vasoconstrictions were significantly higher in the mesenteric arteries (MA) in the caffeine offspring (Table S2). In both the control and caffeine offspring, L-NNA significantly potentiated phenylephrine–induced vasoconstrictions without significant differences between the two groups (Fig. 1B), suggesting that the enhanced sensitivity to phenylephrine in the caffeine offspring was not related to eNOS-mediated relaxation. To determine how vascular sensitivity was enhanced, we tested membrane potential (*E*_m_) for vascular re-activity in MA myocytes. As shown in Fig. 1C, the resting *E*_m_ was the same between the control (−45.67 ± 2.96 mV) and caffeine group (−44.67 ± 4.05 mV). Phenylephrine was more effective in depolarizing the myocytes in the caffeine offspring (Δ 35.67 ± 1.45 mV) than that in the control (Δ 28.04 ± 2.31 mV), suggesting that membrane depolarization might be involved in mechanisms for over re-activity of arterioles in the offspring exposed to prenatal caffeine.

### Reduced LTCCs currents and α_1c_ protein expression in the MA myocytes

**Figure 2:**
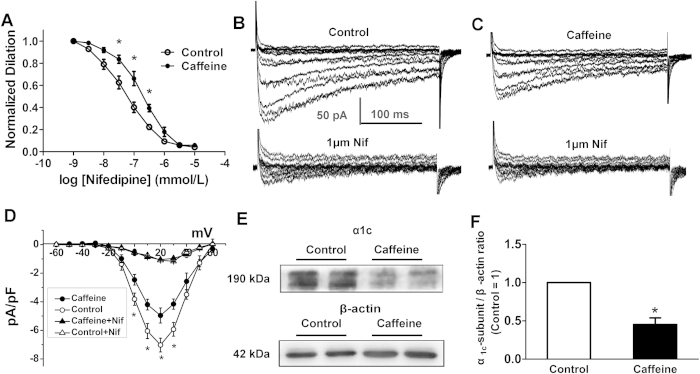
The effects of prenatal caffeine on L-type calcium channels (LTCCs) in mesenteric arteries and myocytes. (**A**) Nifedipine (Nif) concentration-dependently blunted KCl-induced vasoconstrictions (n = 7 each group). *P < 0.05. (**B**,**C)** Representative curve showing whole cell inward currents in absence (top) and presence (bottom) of nifedipine (10^−6^ mol/L) in the control (**B**) and caffeine (**C**) group. (**D)** Current-voltage (I-V) relationships for LTCC currents in the offspring (n = 24 cells, 7 animals/each group). (**E**) Representative blots for α_1c_-subunit of LTCCs in offspring MA. (**F**) The LTCC α_1c_ expression normalized to the control (n = 6 each group). Gels were treated under the same experimental conditions with the control and experimental groups treated together.

Voltage-gated Ca^2+^ channels play a central role in the regulation of vascular tone by membrane potential. L-type voltage-gated Ca^2+^ channels (LTCCs) appear to be dominant in most vascular muscle cells^12^. To determine the hypothesis that an augmented LTCC function may contribute to increased vasoconstrictions and the corresponding myocyte *E*_m_ in the caffeine offspring, we tested KCl-induced MA constrictions with or without LTCCs selective inhibitor, nifedipine (10^−9^ ~ 10^−5^ mol/L). As shown in Fig. 2A, nifedipine dose-dependently inhibited KCl-stimulated constrictions. The inhibited levels were greater in the control. Further electrophysiological data showed that the nifedipine sensitive currents in MA myocytes were weaker in the caffeine group than that in the control (−4.17 ± 0.36 pA/pF vs. −7.03 ± 0.48 pA/pF, Fig. 2B–D). Western blots showed a reduction of α_1C_ protein in the MA of the caffeine group (Fig. 2E,F), indicating that the increased contraction to phenylephrine in the MA of the caffeine offspring may not be attributed to the intrinsic change in functions of LTCCs.

### Altered BK_Ca_ channel function in buffering phenylephrine-induced vessel contraction in prenatal caffeine treated offspring

**Figure 3:**
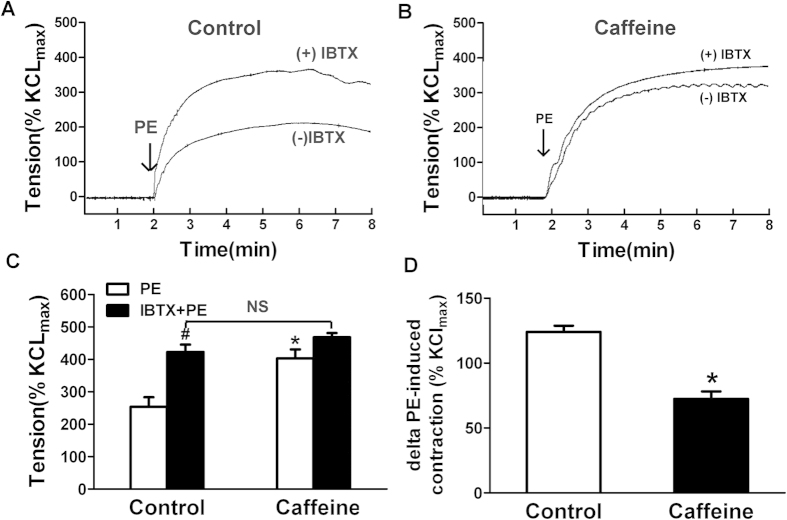
The effect of BK_Ca_ channel on PE-induced contractions of MAs from control and caffeine. (**A**,**B)** The effect of iberiotoxin (IbTX) on PE-induced contraction in MAs from the control (**A**) and caffeine offspring (**B**). (**C**) Statistic diagram for the effect of IbTX pre-treatment on PE-induced vessel contractions. (**D**) The maximal increase of PE-induced vessel contraction (ΔPE). ^#^p < 0.05, compared with PE-induced tension in the same group; *p < 0.05, compared with control.

It is known that the efflux of K^+^ via BK_Ca_ channel activation can be used to counteract pressure- or chemical-induced depolarization and vasoconstriction^13^. In testing whether there was a dysfunction in BK_Ca_ channels in the MA myocytes from the caffeine offspring, we used iberiotoxin (IbTX), a selective BK_Ca_ inhibitor, to determine the BK_Ca_ channel function in buffering phenylephrine-induced vessel contraction. Figure 3 shows the effects of BK_Ca_ channel inhibition on phenylephrine-induced vessel contraction. After BK_Ca_ channel blocked by IbTX (10^−8^ M), the phenylephrine-induced vessel contraction was significantly enhanced ~66.8% (control: from 253.98 ± 29.14 to 422.92 ± 22.98%K_max_), whereas no significant increase can be observed in caffeine offspring (Fig. 3C). The Δphenylephrine-induced vessel contraction after the IbTX treatment in the control was greater than that in the caffeine group, indicating that the contribution of BK_Ca_ in maintaining vascular basal tone was significantly decreased by prenatal caffeine (Fig. 3D). In the presence of IbTX, there were no significant differences in phenylephrine-induced contractions of MAs between the control and caffeine offspring (Fig. 3C). Because the properties of LTCCs was unaltered (Fig. 2), caffeine-mediated enhancements of phenylephrine-induced contractions in MAs should be mainly attributed to the dysfunction of BK_Ca_ channel-mediated relaxation/feedback regulation.

### Reduced BK_Ca_ currents and STOCs activity in the MA myocytes

**Figure 4:**
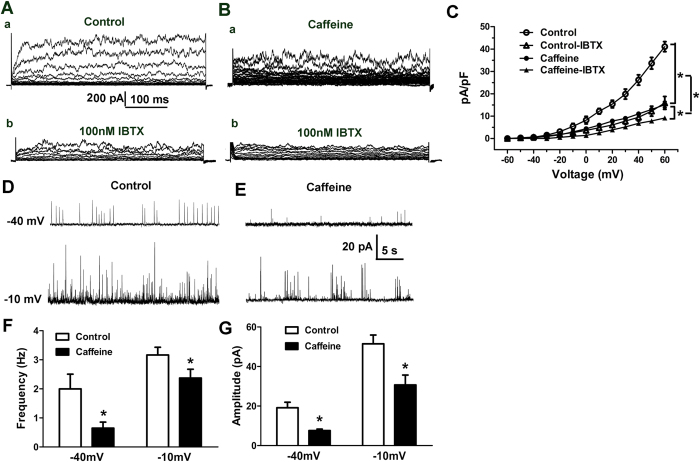
Whole cell BK_Ca_ currents and STOCs in offspring mesenteric arteries. (**A)** whole-cell K^+^ currents measured during depolarizing voltage steps. (**B)** Inhibition of whole-cell K^+^ currents by iberiotoxin (IbTX, 10^−7^ mol/L). (**C**) Mean current density versus voltage plot, in absence or presence of IbTX in myocytes from the control (n = 20 cells, 6 animals) and caffeine group (n = 21 cells, 7 animals). (**D–G)** The effect of prenatal caffeine on spontaneous transient outward currents (STOCs) activity in myocytes. (**D–E**) Representative recordings of STOCs at a holding potential of −40 mV and −10 mV (n = 24 cells, 7 animals/each group). Bar plots summarizing STOCs frequency (**F**) and amplitude (**G**). *P < 0.05, control vs. caffeine.

As shown in Fig. 4A–C, BK_Ca_ currents were significantly reduced in the caffeine group. Since STOCs are hyper-polarizing BK_Ca_ currents against vasoconstriction^14^, we tested activity of STOCs in myocytes under perforated whole-cell patch-clamp conditions. The test potentials were step-wise increased from a holding potential (HP) of −60 mV by 10 mV increments to −10 mV. At membrane potentials higher than −50 mV, STOCs were observed in both the control and caffeine group. There was a voltage-dependent increase in STOCs activity, the increased amplitude and frequency of STOCs were weaker or less in the caffeine group at the same HP. For example (Fig. 4D–G), at a holding potential of −40 mV, activity of STOCs in the caffeine group was significantly reduced. Amplitude of STOCs in the caffeine group was decreased by 60.46% (control:19.12 ± 2.83 pA vs. caffeine: 7.56 ± 0.76 pA). Concomitantly, frequency of STOCs was decreased by 67.34% (control: 1.99 ± 0.50 Hz vs. caffeine: 0.65 ± 0.21 Hz). At higher depolarized *E*_m_, e.g, −10 mV, the frequency and amplitude of STOCs were still reduced in the caffeine group. Thus, a dysfunction of BK_Ca_ channels, especially the diminished STOCs activity, could be counted for a higher phenylephrine-induced depolarization in the smooth muscle cells (SMCs) from the caffeine offspring.

### Attenuated calcium/voltage sensitivity of BK_Ca_ channels resulted from the down-regulation of BK_Ca_ β1-subunits

**Figure 5:**
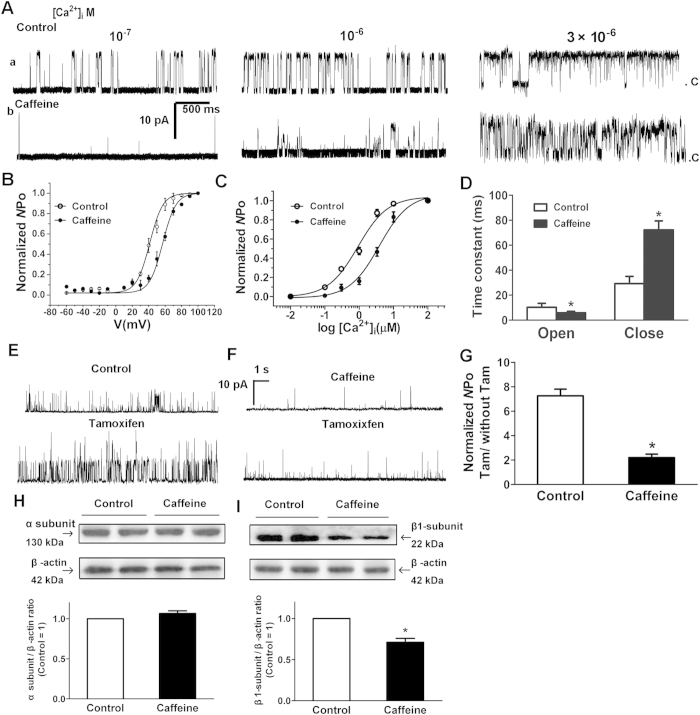
The effects of prenatal caffeine on biophysical properties of BK_Ca_ channels on myocytes from mesenteric arteries (MA). (**A**) Representative single BK_Ca_ channel records in inside-out patches (HP = + 40 mV) from the control (a) and caffeine (b) myocytes exposed to increasing [Ca^2+^]_i_. (**B**) Voltage dependence of BK_Ca_ channels in [Ca^2+^]_i_ at 10^−6^ mol/L. The line was drawn according to the best fits with the Boltzmann equation. (**C**) The effect of prenatal caffeine on Ca^2+^ sensitivity of BK_Ca_ channels. The data points were fitted with the Hill equation to obtain the calcium concentration necessary to open half of the channels (K_d_) and the Hill coefficient (*η*^H^). (**D**) Summary of time constant for open and close state of BK_Ca_ channels ([Ca^2+^]_i_ = 10^−6^ mol/L, HP = + 40 mV; n = 30 cells, 7 animals/each group). (**E–F**) Representative single BK_Ca_ channel recording in inside-out patches (HP = + 40 mV, [Ca^2+^]_i_ = 10^−7^ mol/L) from the control (**E**) and caffeine (**F**) myocytes exposed to 10^−6^ mol/L tamoxifen (Tam). (**G**) Bar plot summarizes the mean ± SEM fold change in the Normalized NPo of BK_Ca_ channels after the application of Tam. (n = 30 cells, 7 animals/each group). (**H–I)** Protein expression of BK_Ca_ α (**H**) and β1-subunit (**I**). The α and β1-subunit expression in the caffeine MA was presented relative to control vessels (n = 6 each group). *P < 0.05, control vs. Caffeine. Gels were treated under the same experimental conditions with the control and experimental groups treated together.

Localized and transient elevations in cytosolic Ca^2+^ (Ca^2+^ sparks), caused by Ca^2+^ release from sarcoplasmic reticulum, are thought to trigger opening of BK_Ca_ channels resulting in STOCs in SMCs^15^. The decreased STOCs activity could be explained by a decreased channel conductance or the number of BK_Ca_ channels activated by a Ca^2+^ spark. As shown in Figure S2A, single BK_Ca_ channel conductance was the same between the two groups (250.27 ± 10.90 vs. 251.93 ± 13.56 pS). The α-subunit selective BK_Ca_ opener, NS-1619 (2 × 10^−5^ mol/L) exerted a similar effect on BK_Ca_ currents in the control and caffeine myocytes (Figure S2B). Moreover, western blot analysis failed to reveal differences in the expression of α-subunits between the control and caffeine group (Fig. 5H). Because α-subunit plays roles in pore-forming in functional BK_Ca_ channels^15^, those results indicated unchanged number of functional BK_Ca_ channels in the caffeine myocytes. Consistent with this, single channel recording demonstrated no differences in the number of BK_Ca_ channels per membrane patch between the two groups (Figure S2C).

Another possible explanation for the reduced STOCs was a decrease in the percentage of sarcoplasmic reticulum (SR) Ca^2+^ release that activated a transient BK_Ca_ currents. Thus, we tested Ca^2+^/voltage sensitivity of BK_Ca_ channels. Figure 5C presents the relationship of NPo normalized to their maximum value against [Ca^2+^]_i_. Both groups showed an enhanced NPo of BK_Ca_ channels with increasing Ca^2+^ concentrations, while the Po-calcium curve for the caffeine group was right shifted with an increased *K*_d_ value (control vs. caffeine: 0.92 ± 0.06 vs. 3.85 ± 0.06). There was no significant difference of the Hill coefficient between both groups (1.05 ± 0.03 vs. 1.08 ± 0.04). Po-voltage relations fitted by Boltzmann equation also were determined at 10^−6^ mol/L [Ca^2+^]_i_ (Fig. 5B). There was a significantly right shift for BK_Ca_ channels in the caffeine offspring. The half activation voltage (V_1/2_) was 47.29 ± 2.35 mV (Control) vs. 57.08 ± 1.79 mV (Caffeine). Those data showed both the calcium and voltage sensitivity of BK_Ca_ channels were attenuated in the caffeine MA.

Ca^2+^/voltage sensitivity and kinetic stability of BK_Ca_ channels is known to be dependent on presence of β1 accessory subunits^16^. A down-regulation of β1-subunits could explain the changes observed in the caffeine group. Tamoxifen could markedly increase the Po of BK_Ca_ channels when they were associated with β1 subunits^17^. Application of 10^−6^ mol/L tamoxifen (10^−7^ mol/L [Ca^2+^]_i_) evoked approximately 7.31-fold of increase in the Po of BK_Ca_ in the control, while only 2.21-fold in the caffeine group (Fig. 5E–G). The expression of BK_Ca_ β1 subunits was reduced by 29.30% in the caffeine group, while α subunits were unchanged (Fig. 5H–I), showing an altered ratio of α/β subunits in the caffeine group. Since the α/β stoichiometry underlies the stability of BK_Ca_ channel opening^18^, if there was a decrease in the coupling ratio of α/β subunits, BK_Ca_ channel open times could be reduced. We compared the open times of BK_Ca_ channels between the control and caffeine MA myocytes by constructing open time histograms at + 40 mV with 10^−6^ mol/L [Ca^2+^]_i_. Histograms were fitted with a single exponential function (Fig. 5D). BK_Ca_ channels from the caffeine myocytes showed shorter open time (τ_open_ = 5.86 ± 1.23 ms) than those from the control (τ_open_ = 10.26 ± 3.24 ms).

### Decreased functional and molecular RyRs in the MA myocytes

It is well established that transient Ca^2+^ increase/Ca^2+^ sparks via ryanodine receptor Ca^2+^ release channels (RyRs) activates BK_Ca_ channels in generating STOCs and modulating vascular tone^19^. Thus, in spite of an intrinsic alteration in biophysical properties of BK_Ca_ channels, the attenuated STOCs activity observed in the caffeine group could be resulted from the decreased Ca^2+^ release from the ryanodine sensitive stores.

**Figure 6:**
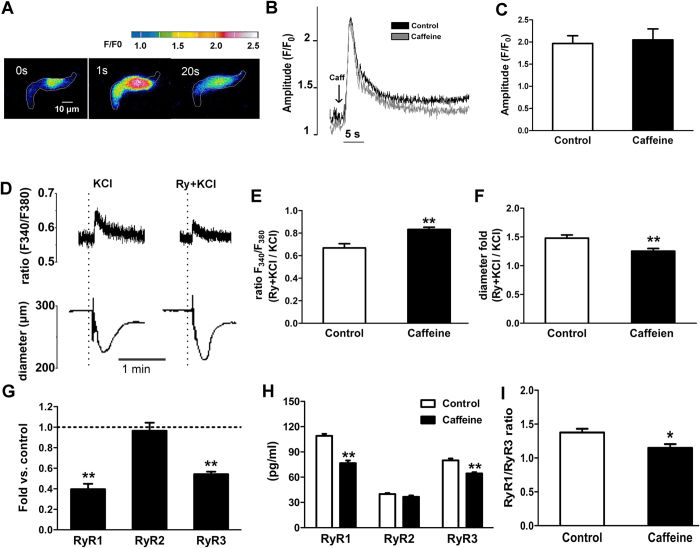
The effects of prenatal caffeine on functional and molecular ryanodine receptors in myocytes from offspring mesenteric arteries (MA). (**A**) Representative single cell images showing Ca^2+^ fluorescence transients at given time points elicited in a MA myocyte. The edge of cells indicated by white line. The images are color coded as indicated by the bar. (**B**,**C**) Representative traces (**B**) and statistics (**C**) of Ca^2+^ transients elicited by application of 10^−2^ mol/L caffeine (n = 20 cells, 7 animals/each group). (**D**) Representative record simultaneously showing Ca^2+^ mobilization (upper) and KCl-induced vasoconstrictions (bottom) in the MA. Vessels were first contracted with 60 mM KCl, following washing and re-equilibration, re-contracted by KCl in presence of ryanodine (Ry + KCl). (**E**,**F**) Summary of the fold change in the KCl-induced Ca^2+^ transients (**E**) and diameter change (**F**) after the application of ryanodine (n = 7–10 each group). (**G**,**H**) mRNA (**G**) and protein (**H**) expression of ryanodine receptor isoforms, RyR1, RyR2, and RyR3. mRNA level of control were set to 1.0. (**I**) Ratio of RyR1/RyR3. *P < 0.05, **P < 0.01 *control vs. caffeine. n = 6 each group.

Potassium-mediated depolarization is a common way to increase Ca^2+^ release by opening of RyRs in the SR, which increases subsarcolemmal Ca^2+^ concentrations^20^. We then focused on global Ca^2+^ responses to K^+^. Figure 6D–F shows the influence of 60 mM KCl and ryanodine on global Ca^2+^ responses and the corresponding arterial diameter in the same pressurized MA. The fluorescence ratio (F_340_/F_380_), indicating cytosolic Ca^2+^ levels^21^, was markedly increased by KCl. After RyRs blocked by ryanodine (10^−5^ mol/L) in the same arterial segment, the KCl-induced increase in fluorescence ratio was significantly reduced by 33.14% in the control, where the reduction was significantly less in the caffeine group (16.70%), indicating that the Ca^2+^ release by activation of RyRs could be reduced in the caffeine group (Fig. 6E). Simultaneously recording diameter showed KCl-reduced arterial diameter, the reductive level was enhanced by ryanodine with different folds between the two groups (Fig. 6F). Vasoconstrictions induced by ryanodine were consistent with inhibition of the negative feedback of RyRs-BK_Ca_ /STOCs in SMCs^22^,^23^. The data above demonstrated the decreased Ca^2+^ release through RyRs, weaker STOCs to the stimulation, depolarizing membrane potential, and increased vascular tone, in the MA from the caffeine offspring.

In subsequent experiment, we measured global Ca^2+^ responses to caffeine (Fig. 6A), since this chemical directly activates all RyRs, not like KCl that indirectly activates RyRs by depolarizing plasma membrane^24^. Application of 10^−2^ mol/L caffeine elicited similar Ca^2+^ transients in both groups (Fig. 6B–C), excluding a change in the SR Ca^2+^ store that may be accounted for the altered Ca^2+^ transient through RyRs in the caffeine group.

Because molecular alteration in RyRs is highly related to their responses to depolarization and caffeine^25^,^26^,^27^,^28^, we examined the expression of mRNA and protein of RyR1, RyR2, and RyR3 in the MA (Fig. 6G–H). Unlike RyR1 and RyR3 that displayed nonparallel reduction in mRNA and protein in the caffeine offspring, RyR2 remained unchanged. RyR1/RyR3 ratio was 1.38 ± 0.09 in the control and 1.15 ± 0.13 in the caffeine group (Fig. 6I).

## Discussion

The main findings of the present study include: 1) Prenatal chronic exposure to high dose of caffeine significantly increased phenylephrine-stimulated pressor responses, vascular-activity, and membrane depolarization in the adult offspring; 2) Prenatal caffeine influenced LTCCs functions associated with reduced α_1c_ protein expression in the MA; BK_Ca_ currents were reduced in the caffeine offspring; 3) BK_Ca_ channel function in buffering phenylephrine-induced vascular constriction was impaired by prenatal exposure to caffeine. Perforated patch recording demonstrated a reduced STOCs frequency/amplitude in the caffeine offspring. Single channel recordings revealed degraded biophysical/intrinsic properties of BK_Ca_ channels, including reduced Ca^2+^/voltage sensitivity and stability of open states. Ca^2+^ fluorescence imaging showed a reduced Ca^2+^ release from SR. Molecular biology approaches revealed a decreased expression of BK_Ca_ β1-subunit, RyR1, and RyR3 in the caffeine group.

Prenatal caffeine induced 25.1% IUGR in our experiments. IUGR is widely accepted as a critical cause for increased susceptibility of cardiovascular diseases^29^,^30^. The etiology of IUGR is largely attributed to adverse pregnant environments, including malnutrition^31^,^32^, hypoxia^33^, and nicotine exposure^34^. The present study added new important information for the theory of “adult diseases in fetal origins” that chronic intake of higher concentrations of caffeine over almost whole gestation could cause long-term influence on cardiovascular health associated with IUGR.

Our initial ideas proposed that both vascular functions in vasorelaxation and vasoconstriction may be counted for the altered vascular responses to phenylephrine in the caffeine group. However, the data of inhibition of eNOS with L-NNA did not support that NO-dependent vasorelaxation may be involved. Thus, we focused on the increased vessel activity. In the present study, prenatal caffeine significantly increased pressor responses to the classic vasoconstrictor, suggesting an enhanced risk in development of hypertension in life later. Previous studies demonstrated that nicotine exposure in pregnancy increased angiotensin II-stimulated pressor responses in the young offspring^35^ and increased blood pressure (BP) in life later^36^, indicating an increased tendency of hypertension in the offspring exposed to prenatal insults if there were altered pressor responses at the early stage. Another study recently reported that prenatal hypoxia led to a higher basal BP in the rat offspring at 9-months-old rather than at the 3-months-old^37^. Those studies, together with our new finding, support the idea that adverse intrauterine environments may have a long-term impact on BP, leading to premature vascular aging, and contributing to increased risk of cardiovascular events during aging.

To determine mechanisms underlying the increased pressor and vascular tone, we focused on resistance arteries and ion channels on smooth muscle cells. Numerous studies showed physiopathologic basis of vascular disorders involving dysfunction of L-type Ca^2+^ channels, e.g, an up-regulation of LTCCs in the genesis of hypertension and hypercholesterolemia^36^,^38^,^39^. Recently, our colleagues demonstrated that high-sucrose diets in pregnancy altered angiotensin II-mediated pressor responses and vessel tone via PKC/Cav1.2 pathway^40^. In the present study, phenylephrine-mediated maximal MA constriction was significantly higher in the caffeine offspring. However, the contribution by LTCCs in KCl-stimulated vasoconstrictions was decreased. Since both the patch clamp and western blot results showed degraded LTCCs, the phenylephrine-increased vasoconstriction was unlikely dependent on the intrinsic properties of LTCCs. Instead, LTCCs-independent factors such as impaired BK_Ca_ channel functions should be taken into consideration. Our investigation at cell level showed that BK_Ca_ currents/STOCs in the caffeine offspring were markedly reduced. Because STOCs act in hyper-polarizing and relaxing vascular SMCs^14^, loss of STOCs activity could explain the increased MA re-activity in the caffeine offspring. In the present study, an obvious reduction in BK_Ca_ channel Ca^2+^/voltage sensitivity and modulatory β1-subunits should be causes of the reduced STOCs. The BK_Ca_ β1-subunits modify functional properties of pore-forming α-subunits, including increasing channel Ca^2+^ sensitivity, slowing down macroscopic activation, and deactivating kinetics, and decreasing voltage sensitivity^15^. A balanced coupling of both subunits is critical to maintain functional properties. Previous study showed that the β1/α subunit ratio in the MA decreased with aging^41^, thus, prenatal caffeine may lead to premature vascular aging, and contributing to increased risks of cardiovascular diseases.

The mammalian RyRs are encoded by 3 different genes (*RYR1*, *RYR2*, and *RYR3*)^42^. The present study showed presence of all three RyRs subtype mRNA in rat mesenteric arteries. RyR1 and RyR2 are co-partners required for Ca^2+^ release during Ca^2+^ sparks and global Ca^2+^ responses in vascular myocytes, evoked by activation of voltage-gated Ca^2+^ channels. RyR1 and RyR2 appeared functionally capable to couple with BK_Ca_ channels. In contrast to RyR1 and RyR2, Ca^2+^ spark triggering arterial tone is precisely adjusted by RyR3. RyR3 is part of the SR Ca^2+^ spark release unit, and feeds back RyR1 and RyR2 to decrease Ca^2+^ spark frequency. RyR3 can be activated by caffeine and local increases in [Ca^2+^]_i_ under conditions of increased SR Ca^2+^ loading^25^,^43^,^44^. In the present study, we found a decline in mRNA and protein expression in RyR1 and RyR3 following prenatal caffeine. These could be the molecular basis underlying the degraded RyRs functions, leading to a reduced Ca^2+^ release from the SR. Although the decreased RyR3 may augment the Ca^2+^ spark and STOCs, the reduced STOCs activity and global Ca^2+^ responses by depolarizing (60 mM KCl) and caffeine could be resulted from stronger suppression of RyR1 than that of RyR3. To the best of our knowledge, this was the first to show altered RyRs by prenatal insults in exploring cellular mechanisms of vascular diseases in developmental origins.

We initially anticipated that an enhanced depolarization of myocytes following prenatal caffeine would be accounted, at least partially, by the increased LTCCs function. However, that was not the case. The functional roles of LTCCs were not enhanced but rather diminished according to the decreased channel currents and pore-forming α_1c_ expression. We suspected those could be a negative-feedback response to limit active vasoconstrictions in preventing vasospasm. Conductive pore-forming α_1c_ LTCCs are regulated by other three (β, α_2_δ, and γ) auxiliary subunits^45^,^46^. Previous studies showed that β-subunits contributed to basal expression of LTCCs in VSMCs. Studies on β3−*/*− mice demonstrated that vascular SMCs expressed fewer LTCCs^47^,^48^. A recent research on a mouse model of genetic hypertension revealed that there existed a “high activity Ca^2+^ influx mode” in which whole-cell LTCCs currents and α_1c_ -subunit expression were decreased while local Ca^2+^ influx activity increased, resulting in an increased frequency and amplitude of RyRs-mediated Ca^2+^ sparks, acting as a feedback mechanism against the degraded coupling between sparks and STOCs. The molecular mechanism underlying high activity of LTCCs showed an increase in α1/α_2_δ/β2 channel-forming complexes, rather than α1/α_2_δ/β3, in myocytes^49^. Whether the LTCCs were reprogrammed by prenatal caffeine as a contribution of decreased I_L−Ca_ cross linking of sparks and cell-wide [Ca^2+^]_i_ changes to altered membrane potentials deserves further investigation.

Although the STOCs activity at a holding potential near resting *E*_m_ (~−40 mV) (Fig. 4) was weaker in the caffeine offspring, the resting *E*_m_ seemed the same between the two groups. We consider that the decreased LTCCs may compensate for the down-regulated BK_Ca_ channels which tend to depolarize resting *E*_m_. Moreover, a sub-population of highly active channels that dominate resting *E*_m_, such as Kv and Kir channels, together with LTCCs, can determine the unchanged resting *E*_m_. For BK_Ca_ to act in a negative feedback role in limiting increased vasoconstrictions, when membrane depolarized, an increase in BK_Ca_ activity also would be expected. Thus, an abnormal depolarization due to BK_Ca_ dysfunction could be seen obviously under the higher *E*_m_.

Based on those findings, we proposed a model for a mechanistic explanation of enhanced myocyte depolarization and vasoconstrictions observed in the caffeine offspring (Figure S3). Chronic high dose of caffeine in pregnancy may cause a suppressed expression of BK_Ca_ β1 subunits, insensitivity of BK_Ca_ to SR Ca^2+^ release, and increase of *E*_m_. The reduced RyR1, RyR3, and Ca^2+^ release via RyRs influenced BK_Ca_ activation/STOCs, leading to a poor negative feedback of *E*_m_ in limiting vasoconstrictions. On the other hand, the expression of LTCCs was decreased, which probably occurred as a protective mechanism against the increased membrane potential and vascular re-activity. The present work focused on male offpsring only, future projects should consider the female too. Based on previous studies on both male and female offspring, there could be similar and different effects of prenatal insults on offspring between the male and female.

In summary, the present study on resistance arteries was the first to raise the possibility that there is a selective down-regulation of β1 subunits accompanied by nonparallel suppression of RyRs resulting in an impairment of RyRs-BK_Ca_ signaling, which could be counted for the increased pressor responses as risk of hypertension in fetal origins. Significance of the findings includes that daily high doses of caffeine or probably too much high concentrated coffee or tea during pregnancy may be risk in disease development in fetal origins.

## Methods

### Animals

Sprague-Dawley rats were used and the experimental procedures were approved by the Institutional Committee of Soochow University and in accordance with the Guide for the Care and Use of Laboratory Animals (NIH Publication No. 85–23, 1996).

### Measurement of pressor responses

Male adult offspring (5-month-old) were implanted with catheters for recording of BP as described^50^. Two days after surgical recovery, BP was recorded in conscious and unrestrained rats.

### Measurement of vessel tone

Small segments of mesenteric arteries (A3~A4) were isolated as described previously^51^. The rings were equilibrated for 60 min.The vessel constrictions to drugs were evaluated by measuring the maximum peak and expressed as percent of maximal tension achieved to 60 mM KCl (K_max_).

### Electrophysiological measurement

SMCs from small mesenteric arteries were isolated^51^,^52^. STOCs were measured using perforated whole-cell patch-clamp technique. BK_Ca_ single channel currents were recorded from inside-out patches. Whole-cell K^+^ currents were measured in conventional whole-cell patch-clamp configuration. SMCs membrane potential was evaluated as previously described^53^.

### Western blot analysis

Small mesenteric arteries were collected and protein was extracted, estimated, and transferred to the membrane using a routine method.

### [Ca^2+^]_i_ imaging and vessel diameter

The arterial segments were mounted and pressurized in a chamber (Living Systems, Burlington, VT). [Ca^2+^]_i_ in mesenteric arteries was monitored using Ca^2+^ indicator Fura 2-AM (Calbiochem, San Diego, CA, USA), as described previously^54^,^55^,^56^,^57^.

### Sub-cellular Ca^2+^imaging

Isolated SMCs were loaded with Fluo-3 AM. For fluorescence imaging, the cell chambers were positioned on the stage of an Olympus IX81 inverted microscope equipped with a Xenon MT-ARC/XE system and an OBS NN10 CCD camera (Olympus, Japan). Image acquisition and analysis was performed using xcellence rt01 (Olympus, Japan).

### Real-time quantitative PCR

Total RNA was extracted and quantified. RNA samples were reverse-transcribed into cDNA using a routine method.

### ELISA analysis

Mesenteric arteries were homogenized with 20% ethanol in phosphate buffer solution (PBS), then centrifuged at 3000 × g at 4 °C for 5 min. Supernatants were collected for analysis using an ELISA kit (JIMIAN Industrial, Shanghai, China) following the manufacturer’s protocol. Please see supplement online for details.

### Data analysis and statistics

Data are presented as mean ± SEM. *t* test or ANOVA, when appropriate, was used to determine significance of differences among groups. A probability value of <0.05 was considered statistically significant.

## Additional Information

**How to cite this article**: Li, N. *et al.* Chronic fetal exposure to caffeine altered resistance vessel functions via RyRs-BK_Ca_ down-regulation in rat offspring. *Sci. Rep.* **5**, 13225; doi: 10.1038/srep13225 (2015).

## Supplementary Material

Supplementary Information
