# Supplementary material for: Chronic fetal exposure to caffeine altered resistance vessel functions via RyRs-BKCa down-regulation in rat offspring
Source: Sci Rep. 2015 Aug 17;5:13225. doi: 10.1038/srep13225 (PMC4642531; doi:10.1038/srep13225)
Supplement: Supplementary Information [file srep13225-s1.pdf]

ONLINE SUPPLEMENT

Chronic fetal exposure to caffeine altered resistance vessel functions via RyRs-BK_{Ca} down-regulation in rat offspring

Na Li[1#], Yongmei Li[1#], Qinqin Gao[1], Dawei Li[1], Jiaqi Tang[1], Miao Sun[1], Pengjie Zhang[1], Bailin Liu[1], Caiping Mao[1] and Zhice Xu[1,2,*]

[1]Institute for Fetology, First Hospital of Soochow University, Suzhou, China

[2]Center for Perinatal Biology, Loma Linda University, California, USA

[#], These authors contributed equally to this work.

Materials and Methods

Animals and treatments

Sprague-Dawley rats (Su Pusi Biotech., Suzhou, China) were acclimated for one week before being subjected to experimental conditions. Rats were allowed free access to standard food and water. Two female rats were placed together with one male rat. The day at which the evidence of mating (i.e., vaginal plug or vaginal smear with sperm cells) was observed was designated as gestational day (GD) 0.5. Saline (control group:16 mothers) or caffeine (caffeine group: 16 mothers; 20 mg/kg; twice a day) was subcutaneously injected daily to pregnant mothers from GD 3.5 to 19.5. On GD20.5, the rats (~380-400g) were anesthetized with sodium pentobarbital (100 mg/kg; Hengrui Medicine, Jiangsu, China). After cesarean section delivery, fetal weight was measured (the litter size of each pregnant rat was 9 to 13, eight mothers each group). All fetal rats/each group were calculated for *in utero* growth restriction (IUGR) rate using the reported criteria (IUGR was diagnosed when the body weight of each individual animal from the treated group was two standard deviations less than the mean body weight of the control group)[1]. Natural delivery was allowed for other pregnant rats. The male offspring (35~40 form 7~8 mothers/each group) were studied at 5-month-old.

Measurement of pressor responses

Sixteen offspring rats (n=8 form 7 mothers per group) were implanted with catheters in their femoral arteries as described[2] under anesthesia with a mixture of ketamine (75mg/kg) and xylazine (10mg/kg; i.p., Hengrui Medicine, Jiangsu, China). Two days after surgical recovery, blood pressure was recorded in conscious and unrestrained rats. Offspring were administered with phenylephrine (10μg/kg in 0.2ml saline) subcutaneously via implanted catheter, and pressor response was monitored for 60 minutes using the Power-Lab system and software (AD Instruments, Bella Vista, Australia).

Measurement of vessel tone

Small segments of mesenteric arteries (A3~A4) were isolated in Krebs–Henseleit solution containing (mmol/L) 125 NaCl, 13.5 $NaHCO_3$, 4.6 KCl, 1.2 NaH_2PO_4, 1.2 $MgCl_2$, 2.5 $CaCl_2$, 0.025 EDTA, and 10 glucose; pH 7.4 with NaOH, mounted on a myograph system

(Danish Myo Technology, Midtjylland, Denmark), bubbled with 95% O_2-5% CO_2 at 37 ℃[3]. The vessel constrictions to drugs was evaluated bymeasuring the maximum peak and expressed as percentof maximal tension achieved to 60mM KCl (K_{max}).

To examine the effect of inhibition of BK_{Ca} channel on tension, vessels were first contracted with phenylephrine (10^{-5} mol/L). After washing and re-equilibration, the vessel was pre-treatedwith selective BK_{Ca} channel inhibitor iberiotoxin (IbTX,10^{-8} mol/L) for 30 minutes, and then re-stimulated with phenylephrine (10^{-5} mol/L). The increase of phenylephrine-induced vessel constriction was calculated using the following formula:
$[Tension_{(IbTX+PE)}/Tension_{(PE)} - 1] \times 100\%$

To examine the effect of inhibition of L-type calcium channels on vessel tension, nifedipine (10^{-9}~10^{-5} mol/L) was added to the bath 30 minutes before adding KCl. Each vessel was used once, signals were recorded by Power-Lab system with Chart-5 software (AD Instruments, Bella Vista , Australia).

Isolation of SMCs from offspring MA

Mesenteric arteries were enzymatically dissociated to obtain individual SMC as previously described [3,4]. Briefly, MA was placed in Ca^{2+}-free ice-cold physiological solution (PSS) containing (mmol/L): 135 NaCl, 5.6 KCl, 1 $MgCl_2$, 10 HEPES, 10 glucose, 0.42 Na_2HPO_4, 0.44 NaH_2PO_4, and 4.2 $NaHCO_3$ (95% O_2, 5% CO_2 for 40 min, pH 7.3-7.4 with NaOH). They were cut into small pieces (0.5mm in length), and incubated for 38 min at 37 ℃ with papain (0.004g/ml, Solarbio, China), dithiothreitol (0.001g/m, BioSHARP, China), and ABV (0.002g/ml, BioSHARP, China). Then, tissue was washed 3 times with Ca^{2+}-free PSS, and triturated gently using a wide bore pipette to release single cells. Separated MA myocytes were then stored in Ca^{2+}-free PSS at 4℃. Electrophysiological experimentswere performed within 6 h after isolation.

Perforated whole cell recording

Spontaneous transient outward currents (STOCs) were measured using perforated whole-cell patch-clamp technique. The bath solution contained (mmol/L): 135 NaCl, 5 KCl, 1 $MgCl_2$, 1.8 $CaCl_2$, 10 glucose, and 10 HEPES (pH 7.4 with NaOH). Patch pipettes (3-5MΩ) were filled with an internal solution containing (mmol/L) 110 potassium aspartate (K-Asp), 30 KCl, 1 $MgCl_2$, 10 HEPES, 0.5 EGTA, 3 Na_2ATP and 0.2 amphotericin B (pH 7.2 with KOH). Holding potential was -60mV. Depolarizing test potentials were gradually increased in 10mV increments from -60 to 0mV. Signals were sampled at 10 kHz, filtered at 2 kHz. Data were captured on-line using a Digidata 1440 interface run under the pClamp 10.2 program (Axon Instruments, Foster City, CA). STOCs were analyzed and plotted using Mini Analysis Program (Synaptosoft, Inc., Decatur, GA). Membrane potential was measured at gap-free mode in perforated whole-cell current-clamp configuration, where the membrane current was clamped at 0 pA[5].

Single channel recording

BK_{Ca} single channel currents were recorded from inside-out patches under symmetrical K^+ (145 mmol/L) at room temperature[3,4]. The pipette (15~20 MΩ) solution consisted of (mmol/L): 100 KCl, 45 K-Asp, 1 EGTA, 10 HEPES, and 5 glucose (pH 7.4 with KOH). The bath solution contained (mmol/L): 45 KCl, 100 K-Asp, 1 EGTA, 10 HEPES, and 5 glucose (pH 7.4 with KOH). Free Ca^{2+} in solution was adjusted to the desired value by adding $CaCl_2$ (determined using WinMax C software, Stanford, CA, USA;

http://www.maxchelator.stanford. edu/). Voltage dependent behavior of the channel open probability (Po) was modeled with the Boltzmann function of the form[4]:

$$Po = 1/\{1+ \exp [-ZF/RT (V-V_{1/2})]\}$$

where $V_{1/2}$ is the voltage of half-maximal channel activation and Kv is the potential needed to produce an e-fold change in Po. The Ca^{2+}-dependent activation was fitted with the Hill equation[4]:

$$Po = [Ca^{2+}]_i\eta^H / (K_d\eta^H + [Ca^{2+}]_i\eta^H)$$

where Po is channel open probability, η^H is the Hill co-efficient, and K_d is the $[Ca^{2+}]_i$ required for half activation.The total number of BK_{Ca} channels in an inside-out patch was determined at a voltage of +40 mV with 10^{-4} mol/L free Ca^{2+} in the bath solution[6].

Currents were sampled at 20 kHz and filtered at 2 kHz with a Bessel filter (8-pole). Continuous recordings of no less than 15,000 ms were used for Po and kinetics analysis. Data acquisition and analysis were carried out using pCLAMP 10.2and Clampfit 10.2 software (Axon Instruments, Foster City, CA).

Conventional whole-cell recording

For measurement of whole-cell K^+ currents, conventional whole-cell configuration was conducted. The composition of bath solution was the same as the bath solution used for perforated patch recording. The pipette (3~5 MΩ) solution contained (mmol/L): 110 K-Asp, 30 KCl, 1 EGTA, 3 Na_2ATP, 0.85 $CaCl_2$, 10 Glucose, and 10 HEPES (pH 7.2, with KOH). Outward K^+ currents were elicited by a series of 500 ms depolarizing voltage steps. Voltage steps were made at 10mV increments to +60mV from a holding potential of -60mV.

In recording L-type calcium channel currents (LTCCs), 20 mmol/L $BaCl_2$ was used as a charge carrier to limit current rundown. The bath solution contained (mmol/L): 20 $BaCl_2$, 125 TEA, 1 $MgCl_2$, 10 HEPES, and 10 glucose (pH 7.3 with TEA-OH). The pipette (3~5 MΩ) solution consisted of (mmol/L): 140 cesium glutamate, 1 $MgCl_2$, 10 HEPES, 10 EGTA, 10 Glucose, and 3 Na_2ATP (pH 7.3 with CsOH). Ba^{2+} current was elicited by 250 ms voltage steps from a holding potential of -60mV to test potentials in the range -50 to +70mV with 10mV increments. Series resistance and total cell capacitance were calculated from uncompensated capacitive transients in response to 10ms hyper-polarizing step pulses (5mV), or obtained by adjusting series resistance and whole-cell capacitance. The whole-cell recordings used for analysis should be with a series resistance <20MΩ, electrode resistances >2 GΩ, leakage current < 100pA. Current densities (pA/pF) were obtained for each cell by normalization of whole cell current to cell capacitance to account for differences in cell membrane surface area. All electrophysiological studies were performed using Axon700B amplifier, pCLAMP 10.2 and Clampfit 10.2 software (Axon Instruments, Foster City, CA).

Western blot analysis

MAs from control and caffeine offspring were collected and stored at -80 ℃. The tissues were homogenized in ice-cold lysis buffer (Beyotime Inst. Biotech, China). The lystates were centrifuged at 4 ℃ for 30 min at 12,000 rpm. Protein concentrations were measured by Bradford assay. Samples with equal protein were loaded and separated on 10% SDS-PAGE (BK_{Ca} α-subunits and LTCC α_{1c}-subunits) or a 15% SDS-PAGE (BK_{Ca} β1-subunits). The membranes were incubated with primary antibodies (Santa Cruz, CA, USA), LTCC

α1c-subunits (1:300), BK_{Ca} α-subunits (1:400), BK_{Ca} β1-subunits (1:300), or β-actin (1:1,000 Beyotime Biotech, China) overnight at 4 ℃. Then, incubated with the secondary antibody (anti-goat for LTCCs α1c-subunits and BK_{Ca} β1-subunits, anti-rabbit for BK_{Ca} α-subunits, anti-mouse for β-actin, 1: 4,000, Beyotime Biotech, China). Immunosignals were visualized using chemiluminescence detection (Amersham Biosciences, Piscataway, NJ, USA) and the UVP imaging system (EC3-Imaging-System, Upland, USA). The ratio of band intensity to β-actin was obtained to quantify the relative levels of protein expression.

$[Ca^{2+}]_i$ imaging and vessel diameter

Small mesenteric arteries were dissected under a dissecting microscope. The arterial segments (~200 μm indiameter) were mounted and pressurized in a chamber (Living Systems, Burlington, VT). Vascular intracellular Ca^{2+} concentrations ($[Ca^{2+}]_i$) were measured in the same tissues loaded with the Ca^{2+} indicator Fura 2-AM, as described previously[7,8]. The vessels were pressurized to 45 mm Hg, which was considered the optimum pressure[9]. Arterial diameter and Ca^{2+} signal were recorded using SoftEdge Data Acquisition Subsystem system (IonOptix, Milton, MA) as reported[6,7].

Sub-cellular Ca^{2+} imaging

Isolated SMCs were loaded with Fluo-3 AM (10 μmol/L, Invitrogen, USA.) for 30 min and then washed three times with Ca^{2+} free PSS. Myocytes were then washed with Ca^{2+} bathing PPS and used for fluorescence intensity recordings after a stabilizing period of 10 min. All measurements were performed for 15-45 min following the stabilizing period. For fluorescence imaging, the cell chambers were positioned on the stage of an Olympus IX81 inverted microscope equipped with a Xenon MT-ARC/XE system and an OBS NN10 CCD camera (Olympus, Japan). The microscope was equipped with an immersion objective lens (60×, NA 1.42; PlanApo). The typical image size was 0.11×0.1468 mm (height×width). Image acquisition and analysis were performed using xcellence rt01 (Olympus, Japan). Global Ca^{2+} responses were acquired at roughly one image per 350ms. For analysis of changes in Ca^{2+}-related fluorescence, a region of interest (ROI) was drawn alone the edge of cells. For presentation purposes, the fractional fluorescence intensity was calculated as F/F0=F -baseline/F0-baseline, where baseline is the intensity from a region of interest with no cells, F is the fluorescence intensity for the region of interest, and F0 is the fluorescence intensity during a period from the beginning of the recording when there was no Ca^{2+} activity.

Real-time quantitative PCR

Total RNA was extracted immediately from freshly isolated arterioles, using the RNAiso plus Trizol (Takara, Japan) according to the manufacturer’s instruction，and was quantified by measuring its absorbance at 260/280 nm wavelength. Equal amounts of RNA samples were reverse-transcribed into cDNA using 1st strand cDNA Synthesis kit (Takara). Real-time quantitative PCR was performed using iCycler Thermal Cycler (Bio-Rad, USA). Primers were designed to amplify RyR1 (forward primer: 5’-TCTTCCCTGGAGACTGT-3’; reverse primer: 5’-TGGGAGAAGGCACTTGAGG-3’), RyR2 (forward primer: 5’-ACAACCCAAATGCTGGTCTC-3’; and reverse primer: 5’-TCCGGTTCAGACTTGGTTTC-3’), RyR3 (forward primer: 5’-CTGGCCATCATTCAAGGTCT-3’; reverse primer: 5’-GTCTCCATGTCTTCCCGTA-3’), GAPDH (forward primer: 5’-CCAGGTTGTCTCCTGTGACTTC-3’; and reverse:

5’-ACCAGGAAATGAGCTTCACAAA-3’). The reaction mix contained 12.5 µl 2×SYBR Green MAter mix (Takara), 0.5 µl forward primer, 0.5 µl reverse primer,1.0 µl cDNA, and dd water to a total volume of 25µl. The PCR conditions were as follows: an initial 95 ℃ for 3 min followed by 40 cycles of 95 ℃ for 15s, 58 ℃ for 15s, 68 ℃ for 20s. The relative gene expression (RGE) was calculated as RGE = 2 − ΔΔCt, and the relative expression level was determined on the basis of the comparative ΔΔCt method using the reverse transcription products from the control group as the calibrator.

ELISA analysis

Mesenteric arteries were homogenized with 20% ethanol in phosphate buffer solution (PBS), then centrifuged at 3000 ×g at 4 ℃ for 5 min. Supernatants were collected for analysis using an ELISA kit (JIMIAN Industrial, Shanghai, China) following the manufacturer’s protocol. Briefly, 50µl standard sample was added into the standard sample well, containing 40µl sample dilution and 10µl testing sample (final dilution is 5-fold). The antigen-coated wells were then incubated for 30 min at 37°C, following a five times’ washing with buffer. Then, the antigen-coated wells were incubated with 50µl of HRP-Conjugate reagent for 30min at 37 ℃. The unbound antibodies were washed away with washing buffer, and then incubated with 50µl of Chromogen Solution A and 50µl of Chromogen Solution B. After incubation for 10 min at 37 ℃ in dark, 50µl of stop solution was added to each sample, and the absorbance at 450 nm was determined. Data were handled in a blind manner.

References

1. Valsamakis G, Kanaka-Gantenbein C, Malamitsi-Puchner A, MAtorakos G. Causes of intrauterine growth restriction and the postnatal development of the metabolic syndrome. *Ann N Y Acad Sci.* **1092**, 138-147 (2006).

2. MAsett MP, Lewis SJ, Stauss HM, Kregel KC. Vascular reactivity and baroreflex function during hyperthermia in conscious rats. *Am J Physiol Regul Integr Comp Physiol.* **279**, R1282-R1289 (2000).

3. Chonglong Wu, Jiayue Li, Le Bo, Qinqin Gao, Zhoufeng Zhu, Dawei Li, Shigang Li, Miao Sun, Caiping Mao and Zhice Xu. High-sucrose diets in pregnancy alter angiotensinII-mediated pressor response and microvessel tonevia the PKC/Cav1.2 pathway in rat offspring. *Hypertension Research*. **37**, 818-23 (2014).

4. Na Li, Yue Shi, Lijun Shi, Yujia Liu, Yanyan Zhang. Effects of aerobic exercise training on large-conductance Ca^{2+}-activated K^{+} channels in rat cerebral artery smooth muscle cells. *Eur J Appl Physiol.* **113**, 2553-2563 (2013).

5. Pucovský V, Bolton TB. Localisation, function and composition of primary Ca(2+) spark discharge region in isolated smooth muscle cells from guinea-pig mesenteric arteries. *Cell Calcium*. **39**, 113-29 (2006).

6. Cheng X, Jaggar JH. Genetic ablation of caveolin-1 modifies Ca2+ spark coupling in murine arterial smooth muscle cells.*Am J Physiol Heart Circ Physiol.* **290**, H2309-2319 (2006).

7. Kip SN, Hunter LW, Ren Q, Harris PC, Somlo S, Torres VE, Sieck GC, Qian Q. $[Ca^{2+}]_i$ reduction increases cellular proliferation and apoptosis in vascular smooth muscle cells: relevance to the ADPKD phenotype. *Circ Res*. **96**, 873-880 (2005).

8. Xiao D, Buchholz JN, Zhang L. Pregnancy attenuates uterine artery pressure-dependent vascular tone: role of PKC/ERK pathway. *Am J Physiol Heart Circ Physiol.* **290**, H2337-H2343 (2006).

9. Touyz RM, Endeman D, He G, Li JS, Schiffrin EL. Role of AT2 receptors in angiotensin II-stimulated contraction of small mesenteric arteries in young SHR. *Hypertension* **33**, 366-72 (1999).

Supplementary Figures and Tabels

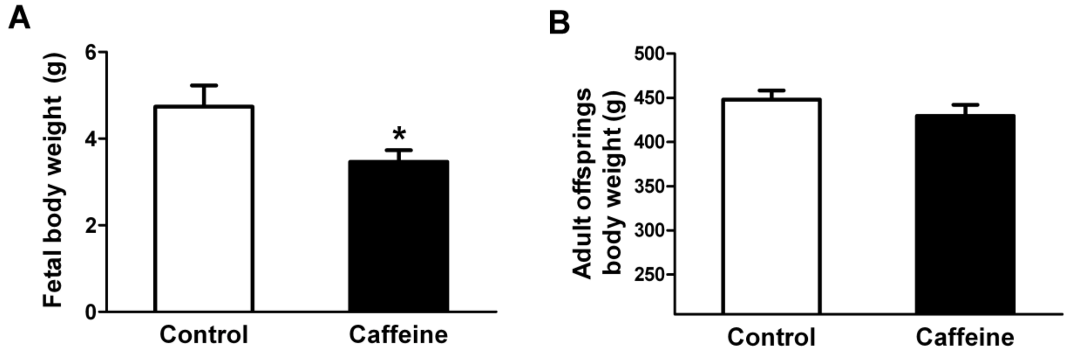

Figure S1. Body weight in fetal (GD 20.5, n=70 fetuses from 6 mothers/group) rats (A) and male offspring (5 month, n=30/group) rats (B). *, $p < 0.05$.

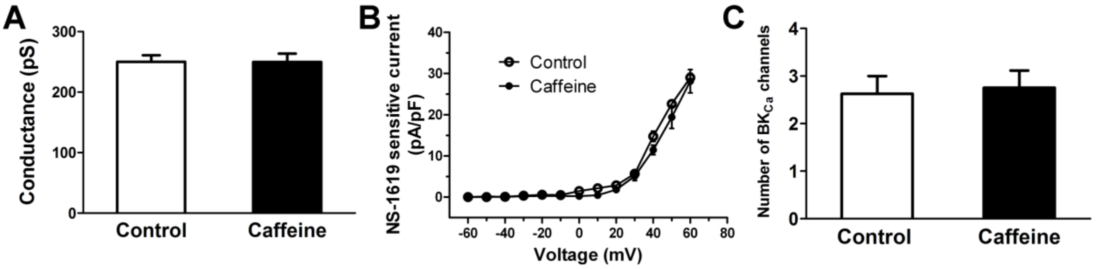

Figure S2.A,Summary of the BK_{Ca} channel conductance in the offspring. **B**, Mean current density activated by NS-1619 versus voltage plot in myocytes between the two groups. **C**, Inside-out patches from the caffeine group contained similar BK_{Ca} channels with patches from the control group. n=23 cells, 6 animals/each group.

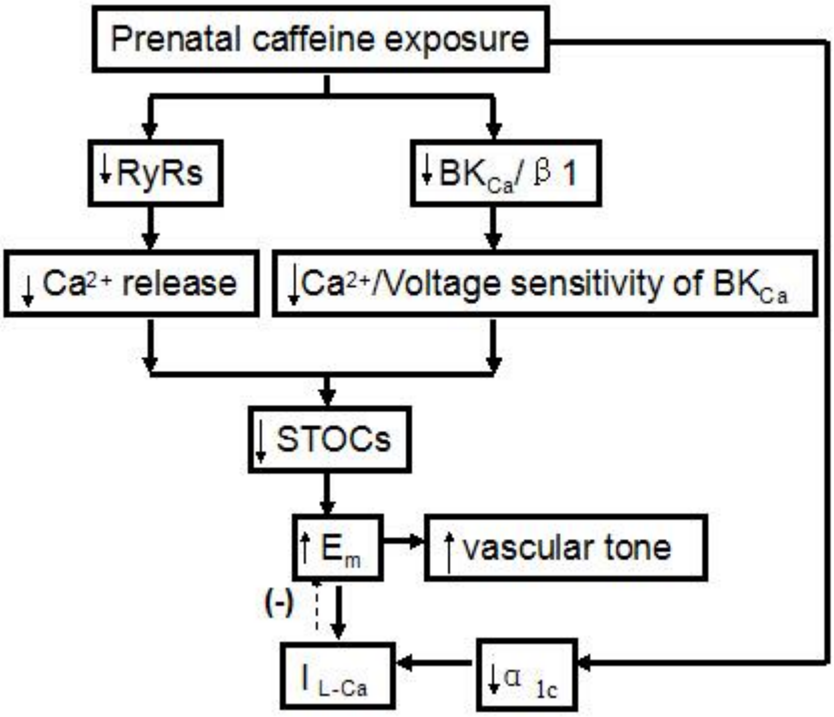

Figure S3. Schematic model for the mechanismsunderlying the altered vascular functions following prenatal caffeine, showing that the degraded BK_{Ca} biophysical properties accompanied withdown-regulated BK_{Ca} β1 subunits, and diminished transient Ca^{2+} release via ryanodine sensitive Ca^{2+} release channels (RyRs) associated with the unparallel down-regulation of the subtypes of RyRs, eliciting reduced spontaneous transient outward currents (STOCs), leading toenhancedmembrane depolarization. The latter prompted increased-Ca^{2+}influx via L-type calcium channels (LTCCs). However, LTCCs currents could be reduced by a decreased expression of α_{1c} subunits, which, in turn,deactivated Ca^{2+}influx and depressed membrane depolarization. Such a compensatory pathway by intrinsic alteration of LTCCs could not completely reverse the higher E_m caused by dis-regulation of STOCs, resulting in an increase of the vessel re-activity and pressor responses to vasoconstrictions.

Table S1. The effect of prenatal caffeine on basal arterial pressure and heart rateinthe adult offspring rats

Animal Group	MAP, mm Hg	SBP, mm Hg	DBP, mm Hg	HR, bpm
Control	115.6 ±2.8	120.4 ±4.3	103.1 ±4.7	355.5 ±19.9
Caffeine	111.7 ±4.5	123.9 ±4.8	99.6 ±4.9	360.6 ±6.0

n=8 each group, MAP: mean arterial pressure; SBP and DBP: systolic and diastolic blood pressure; HR: heart rate.

Table S2. The effect of prenatal caffeine on phenylephrine-mediated vasoconstriction in mesenteric arteries of adult offspring rats in absence or presence of L-NNA

	Control		Caffeine	
Treatment	pD_2	E_{max}	pD_2	E_{max}
-L-NNA	6.28 ±0.10	221.12 ±9.04	6.69 ±0.07*	348.73 ±8.98*
+L-NNA	6.55 ±0.07†	311.47 ±7.98†	6.78 ±0.07	431.87 ±10.68*†

pD_2, -log EC50; Emax, maximal response (% KCl response). n=8 each group.
*, $P<0.05$, control vs. caffeine; †, $P<0.05$, +L-NNA vs. –L-NNA.
